# Axon-somatic back-propagation in detailed models of spinal alpha motoneurons

**DOI:** 10.3389/fncom.2015.00015

**Published:** 2015-02-12

**Authors:** Pietro Balbi, Sergio Martinoia, Paolo Massobrio

**Affiliations:** ^1^Department of Neurorehabilitation, Scientific Institute of Pavia via Boezio, IRCCS, ‘Salvatore Maugeri’ FoundationPavia, Italy; ^2^Department of Informatics, Bioengineering, Robotics, System Engineering (DIBRIS), University of GenovaGenova, Italy

**Keywords:** spinal motoneuron, detailed model, back-propagation, axonal initial segment, sodium channel density

## Abstract

Antidromic action potentials following distal stimulation of motor axons occasionally fail to invade the soma of alpha motoneurons in spinal cord, due to their passing through regions of high non-uniformity. Morphologically detailed conductance-based models of cat spinal alpha motoneurons have been developed, with the aim to reproduce and clarify some aspects of the electrophysiological behavior of the antidromic axon-somatic spike propagation. Fourteen 3D morphologically detailed somata and dendrites of cat spinal alpha motoneurons have been imported from an open-access web-based database of neuronal morphologies, NeuroMorpho.org, and instantiated in neurocomputational models. An axon hillock, an axonal initial segment and a myelinated axon are added to each model. By sweeping the diameter of the axonal initial segment (AIS) and the axon hillock, as well as the maximal conductances of sodium channels at the AIS and at the soma, the developed models are able to show the relationships between different geometric and electrophysiological configurations and the voltage attenuation of the antidromically traveling wave. In particular, a greater than usually admitted sodium conductance at AIS is necessary and sufficient to overcome the dramatic voltage attenuation occurring during antidromic spike propagation both at the myelinated axon-AIS and at the AIS-soma transitions.

## Introduction

Since the early Fifties of the last century, microelectrode intracellular recordings from spinal motoneurons allowed the detailed analysis of the electrophysiological events generated at the soma by an antidromic spike evoked at the distal part of a motor fiber (Brock et al., 1953; Eccles, 1955). This technique, compared to the extracellular recordings which usually collected potentials from a population of neurons, provides much of the most direct information on antidromic invasion of single motoneurons. Thus, it was possible to study the central effect of stimuli applied to motor nerves and antidromically conducted to the soma of spinal alpha motoneurons.

It was evident that, due to the geometric inhomogeneity of the cable conductor which reduces the safety factor of the conduction along the nerve fiber, the antidromic traveling wave was not always able to invade the soma (Eccles, 1955; Goldstein and Rall, 1974; Joyner et al., 1980; Moore et al., 1983).

Classically, three transition regions have been hypothesized to dampen the antidromic spike conduction: (i) the myelinated axon to the unmyelinated axon initial segment (AIS), (ii) the AIS to the soma, and (iii) the profusely branching dendritic tree (Eccles, 1955). In particular, at the AIS to soma transition, a junctional delay or a block of the conduction may happen, due to the increased capacitive load carried by the somato-dendritic region downstream of the antidromically traveling wave (Joyner et al., 1980; Moore et al., 1983). The magnitude of the delay at the AIS to soma transition, or the presence of a complete block at this point depends on morphologic and electrophysiological (mainly the Na^+^ conductance) properties at the transition region (Goldstein and Rall, 1974).

Nowadays, the occurrence of soma invasion has been used in reduced computational models of spinal motoneurons with the aim to indirectly set the values of Na^+^ channel densities onto somatic and AIS membranes (Powers et al., 2012), with the implicit assumption that the somatic invasion always follows an antidromic spike propagation.

In a previous study (Balbi et al., 2014), we developed a reduced model of spinal alpha-motoneuron to investigate the reciprocal interplay between AIS and soma during the antidromic conduction of a spike, and to explore the associated phenomenon of the recurrent discharge.

In the present study, detailed models of spinal motoneurons have been developed and described, taking advantage of both the web-based database NeuroMorpho of somato-dendritic morphologically detailed reconstructions (Ascoli et al., 2007) and a previous model of mammalian nerve fiber (McIntyre et al., 2002). We aimed at exploring the axon-somatic back-propagation in detailed models of spinal motoneurons, with different morphologies of the somato-dendritic part.

In particular, by using detailed models, we derive more plausible and realistic conductances values of the ionic channels responsible of antidromic propagation from axonal to somatic regions.

Finally, the present study also provides a novel yet tentative modeling investigation of the spike propagation at the myelinated-unmyelinated region of the AIS.

## Materials and methods

The proposed models were developed and run in the NEURON v7.3 simulation environment (Carnevale and Hines, 2006). Full details and the source code will be provided as a ModelDB entry (https://senselab.med.yale.edu/ModelDB; accession number: to be defined).

### Geometric properties of the models

The morphologically detailed models are made up of three parts: (a) the soma and a highly detailed three-dimensional dendritic tree, (b) a frustum-shaped axon hillock with the proximal enlarged end attached to the soma, and the opposite end attached to the unmyelinated axonal initial segment (AIS), (c) a myelinated axon (23 mm long) attached to the distal end of the AIS.

#### Soma and dendrites

**Figure 1:**
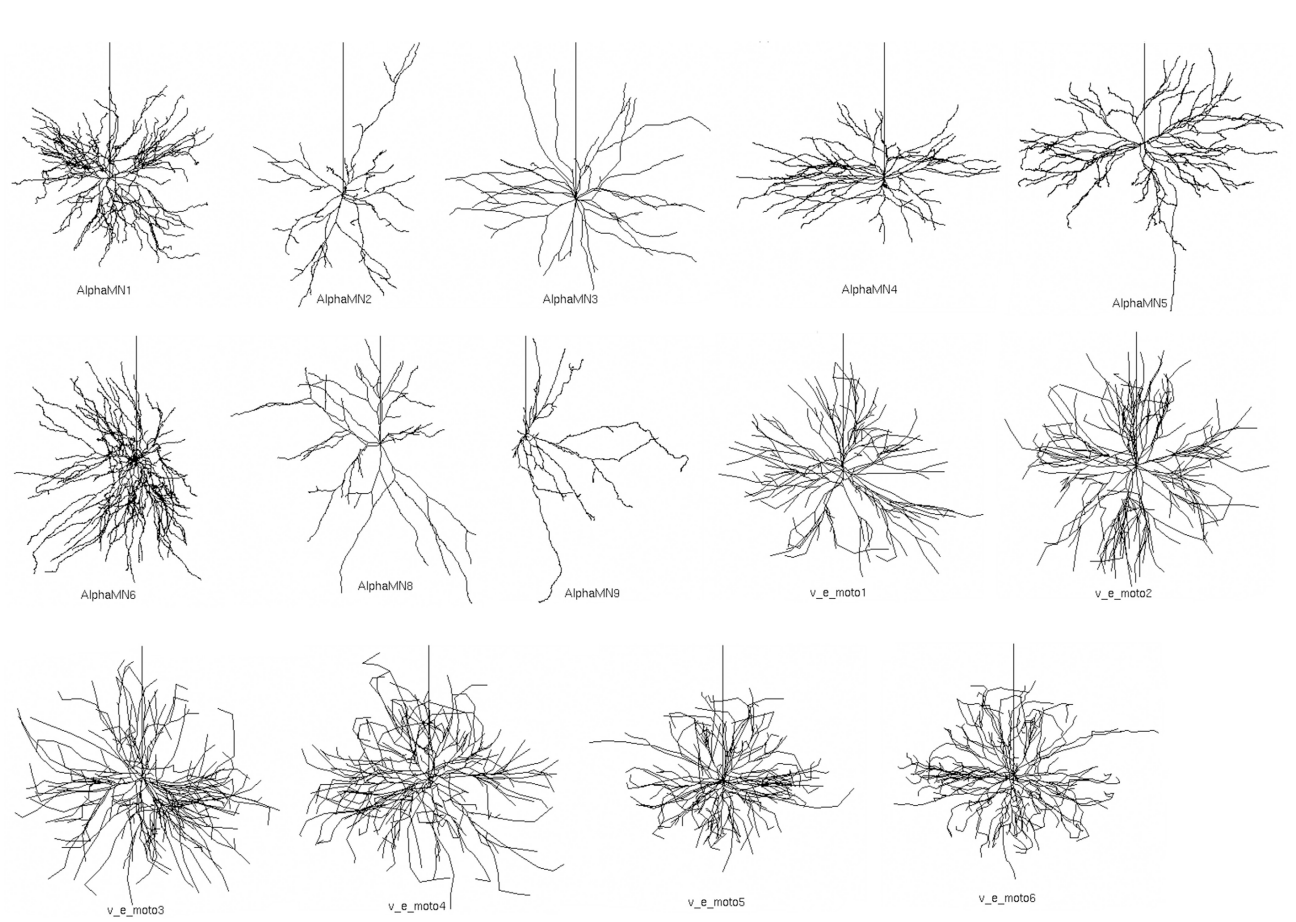
**Fourteen detailed 3D somato-dendritic morphologies of cat spinal alpha-motoneuron imported from NeuroMorpho.org (Ascoli et al., 2007)**. The vertical upward neurite in each neuron represents the proximal part of the axon and was added after importing the somato-dendrite morphologies.

In the simulations, we used reconstructed 3D morphologies of dendritic tree and soma from 14 cat spinal motoneurons (Figure 1), available from the NeuroMorpho database (Ascoli et al., 2007).

The morphologies belong to distinct sets of data from two different works (Cullheim et al., 1987; Alvarez et al., 1998), and morphometric details are provided in Table 1. All 3D morphologies derive from alpha motoneurons of the cat lumbar spinal cord. In NeuroMorpho database reconstruction, the original spherical somata are converted in two cylindrical compartments with identical diameters and lengths; the length of each compartment is half the diameter.

**Table 1:** **Morphologic values of the imported somato-dendritic reconstructions**.

	**NeuroMorpho accession number**	**Number of dendritic sections**	**Number of dendritic compartments**	**Number of primary dendrites**	**Soma diameter (μm)**	**Sum of the diameters of the primary dendrites (μm)**	**Soma surface (μm^2^)**	**Surface of dendrites tree (μm^2^)**	**Ratio of dendrites to soma surface**	**Dendrites mean terminal distance**
AlphaMN1	NMO_00687	398	3118	10	65.02	111.42	13,280	682,012	51.36	1049.45
AlphaMN2	NMO_00688	92	716	8	49.97	87.38	7847	150,243	19.15	736.52
AlphaMN3	NMO_00689	86	540	16	65.64	158.97	13,535	166,343	12.29	456.83
AlphaMN4	NMO_00690	149	1425	13	52.82	89.10	8767	233,642	26.65	829.38
AlphaMN5	NMO_00691	249	2183	11	60.81	101.77	11,616	374,199	32.22	896.49
AlphaMN6	NMO_00692	270	2656	12	63.83	130.10	12,801	489,120	38.21	891.33
AlphaMN8	NMO_00693	91	623	9	57.04	98.42	10,222	161,839	15.83	537.83
AlphaMN9	NMO_00694	80	452	10	69.68	92.30	15,252	138,799	9.10	561.90
v_e_moto1	NMO_00604	254	2414	10	120.00	73.58	45,238	605,023	13.37	1167.14
v_e_moto2	NMO_00605	355	3197	13	95.40	103.84	28,592	803,947	28.12	1104.99
v_e_moto3	NMO_00606	362	3988	12	98.40	77.50	30,419	597,492	19.66	1118.59
v_e_moto4	NMO_00607	370	3964	8	109.20	58.07	37,462	625,236	16.69	1178.81
v_e_moto5	NMO_00608	317	3421	15	97.60	104.36	29,926	708,849	23.69	1166.13
v_e_moto6	NMO_00609	311	3267	11	103.40	98.94	33,589	628,803	18.72	1090.41
Mean		241.71	2283.14	11.29	79.20	98.98	21,324.71	454,681.93	23.22	913.27
SD		119.90	1301.86	2.40	23.55	24.56	12,353.68	241,558.00	11.42	254.13

On the other way round, the dendritic tree is converted in multiple compartments, adopting the convention that a compartment is made by a neurite without ramifications, i.e., the compartment ends when it divides in branches or when it is a terminal ramification. This implies that a single compartment usually has different diameters along its course and that it can run considerably far from its beginning.

Since a poor spatial discretization could lower the performance of the models, we increased the number of dendritic compartments of the reconstructed dendritic tree after importing them in NEURON. In particular, we split in multiple compartments every branch longer than 20 μm, obtaining a nearly 15-fold increase of the average number of dendritic compartments, as displayed in the third and fourth columns of Table 1. The number of compartments in which a section should be divided (spatial discretization) to preserve consistent computational results usually relies on the value of the DC length constant (λ_DC_) of an infinite cylinder with identical anatomical and biophysical properties. A more thorough approach, considering the transient characteristics of the neural signals, takes into account the AC length constant λ_*f*_ computed at a frequency *f* that is high enough for transmembrane current to be primarily capacitative, yet still in the range of frequencies relevant to neural function (Carnevale and Hines, 2006). By using the NEURON built-in function *d_lambda*, we checked that the final spatial grid of the dendritic tree was consistent with the AC length constant rule.

#### AIS and axon hillock

As the reconstructed 3D morphologies are limited to the somato-dendritic part of the spinal motoneurons, we modeled the efferent pathway (i.e., the axon hillock, the AIS and the myelinated axon) based on average measures of cat spinal motoneurons (Conradi, 1969; Burke et al., 1977; Cullheim and Kellerth, 1978; Ulfhake and Kellerth, 1982, 1984; Cullheim et al., 1987). As a result, the cylindrical AIS was divided in 9 compartments, 3 μm wide and 50 μm long. The frustum-shaped axon hillock, formed by 7 compartments, has been modeled 25 μm long and connects the soma with the AIS; its diameter ranges from a value similar to the AIS at one end, to a value similar to the soma at the opposite side, so that a linear transition occurs between the soma and AIS diameters along the axon hillock.

#### Myelinated axon

The myelinated part of the axon has been modeled according to the study of McIntyre et al. (2002), who developed a geometrically and electrically accurate model of mammalian motor nerve fiber. In particular, adopting a double cable structure with a finite impedance myelin sheath, the model details the node of Ranvier, the adjacent paranodal myelin attachment segment, the juxtaparanodal segment and the internodal segment. The geometric details are summarized in Table 2.

**Table 2:** **Geometric parameters of the myelinated axon**.

Node-node separation^a^	1.150
Number of myelin lamella^b^	120
Node length^c^^,^^d^	1
Node diameter^c^^,^^d^	3.3
Length of the paranodal myelin attachment segment^e^	3
Diameter of the myelin attachment segment^b^^,^^e^	3.6
Periaxonal space width of the myelin attachment segment^e^	0.002
Length of the juxtaparanodal segment^e^	46
Diameter of the juxtaparanodal segment^b^^,^^e^	6.9
Periaxonal space width of the juxtaparanodal segment^e^	0.004
Length of the internodal segment^a^	175.2
Diameter of internodal segment^b^	6.9
Periaxonal space width of internodal segment^e^	0.004

*Values expressed in μm. Each internode composed by 6 internodal segments*.

a *Nilsson and Berthold (1988)*.

b *Berthold et al. (1983)*.

c *Rydmark (1981)*.

d *Rydmark and Berthold (1983)*.

e *Berthold and Rydmark (1983)*.

### Biophysical properties

Passive and active properties of the model, derived from previous *in silico* (McIntyre and Grill, 2002; McIntyre et al., 2002; Powers et al., 2012) and *in vivo* (Araki and Terzuolo, 1962; Kernell, 1966; Barrett and Crill, 1974, 1980; Barrett et al., 1980; Zengel et al., 1985; Morales et al., 1987; Fleshman et al., 1988; Clements and Redman, 1989; Yamuy et al., 1992; Liu et al., 1995) studies are provided in Tables 3, 4; the rate functions of the ionic channels, according to the Hodgkin-Huxley formalism (Hodgkin and Huxley, 1952), are described in Supplemental Material.

**Table 3:** **Electrical conductances of soma, dendrites, AIS, and axon hillock**.

**SOMA AND PROXIMAL DENDRITES**	
Maximum fast Na^+^ conductance (g_Na_), mS/cm^2^	15:0.15
Maximum delayed rectifier K^+^ conductance (g_Kdr_), mS/cm^2^	35:0.3
Maximum persistent Na^+^ conductance (g_Nap_), mS/cm^2^	2:0.015
Maximum Ca^2+^ activated K^+^ conductance (g_K(Ca)_), mS/cm^2^	10:0
Maximum high-threshold Ca^2+^ conductance (g_CaH_), mS/cm^2^	0.008:0
Maximum hyperpolarization-activated mixed cation conductance (g_H_), mS/cm^2^	0.1325
Leakage conductance (g_L_), mS/cm^2^	4.4:0.072
**MEDIUM AND DISTAL DENDRITES**	
Maximum fast Na^+^ conductance (g_Na_), mS/cm^2^	0.15
Maximum delayed rectifier K^+^ conductance (g_Kdr_), mS/cm^2^	0.3
Maximum persistent Na^+^ conductance (g_Nap_), mS/cm^2^	0.015
Maximum hyperpolarization-activated mixed cation conductance (g_H_), mS/cm^2^	0.1325
Leakage conductance (g_L_), mS/cm^2^	0.072
Maximum low-threshold Ca^2+^ conductance (g_CaL_), mS/cm^2^^*^	0.28
Maximum Ca^2+^ activated K^+^ conductance (g_K(Ca)_), mS/cm^2^^*^	0.16
**AXON HILLOCK**	
Maximum fast Na^+^ conductance (g_Na_), mS/cm^2^	15:300
Maximum delayed rectifier K^+^ conductance (g_Kdr_), mS/cm^2^	35:400
Maximum persistent Na^+^ conductance (g_Nap_), mS/cm^2^	2:10
Leakage conductance (g_L_), mS/cm^2^	4.4
**INITIAL SEGMENT**	
Maximum fast Na^+^ conductance (g_Naf_), mS/cm^2^	300
Maximum delayed rectifier K^+^ conductance (g_Kdr_), mS/cm^2^	400
Maximum persistent Na^+^ conductance (g_Nap_), mS/cm^2^	10
Leakage conductance (g_L_), mS/cm^2^	4.4

^*^Only onto medium dendrites.

**Table 4:** **Myelinated axon electrical parameters**.

Nodal capacitance (*C_n_*), μF/cm2	2
Internodal capacitance (*C_i_*), μF/cm^2^	2
Myelin capacitance (*C_my_*), μF/cm^2^	0.1
Axial intracellular resistivity (*ρ_i_*), Ω cm	70
External resistivity (*ρ_o_*), Ω cm	70
Node leakage conductance (*g_n_*), mS/cm^2^	1
Paranode leakage conductance (*g_p_*), mS/cm^2^	1
Juxtaparanode leakage conductance (*g_j_*), mS/cm^2^	0.1
Internode leakage conductance (*g_i_*), mS/cm^2^	0.1
Myelin leakage conductance (*g_my_*), mS/cm^2^	1
Maximum fast Na^+^ conductance (*g_Naf_*), mS/cm^2^	880
Maximum slow K^+^ conductance (*g_K_*), mS/cm^2^	400
Maximum persistent Na^+^ conductance (*g_Nap_*), mS/cm^2^	4.4
K^+^ equilibrium potential (*E_K_*), mV	−77
Na^+^ equilibrium potential (*E_Na_*), mV	+50
Leakage equilibrium potential (*E_L_*), mV	−72

*Electrical parameters according to McIntyre et al. (2002)*.

#### Passive properties

All the neuronal segments have been modeled with axial and external resistivity set at 70 Ω · cm. Specific capacitance has been set at 1 μF/cm^2^ (Barrett and Crill, 1974; Gentet et al., 2000), except in myelin, where a value of 0.1 μF/cm^2^ has been adopted, and in nodal and internodal axonal membrane, where it has been set at 2 μF/cm^2^ (McIntyre et al., 2002).

The Na^+^ equilibrium potential has been set to +50 mV, the K^+^ to −77 mV, while the Ca^2+^ equilibrium potential dynamically changed depending on the variations of internal and external ion concentrations. The Ca^2+^ dynamics were represented by a simple, first-order process representing influx through Ca^2+^ channels and decay with a single time constant (see Supplemental Material for details).

#### Active properties

***Soma and proximal dendrites***. We considered proximal dendrites those parts of the primary dendrites extending from the soma along a distance equal to the 10% of the average dendritic length for each neuron model. The soma and the proximal dendrites include conductances representing non-linear fast Na^+^, persistent Na^+^, delayed rectifier K^+^, hyperpolarization-activated mixed cation, high-threshold Ca^2+^, and Ca^2+^-activated K^+^ channels as well as a linear leakage conductance (Table 3). The somatic Ca^2+^-activated K^+^ channel is responsible for the after-hyperpolarization (AHP). Along the proximal dendrites, each conductance linearly decreases to the respective value set in the distal part of the dendritic tree, except for the hyperpolarization-activated conductance, which has the same value in soma and dendrites.

As regard as the sodium currents, we decided to adopt separate models for the transient and the persistent component of the Na^+^ channels. In real neurons the persistent sodium current is not provided by a separate channel, but it is a feature of the Nav1.6 channel electrophysiological behavior. However, accurate representation of both of these components requires a multi-state kinetics model (Kuo and Bean, 1994). In addition, a series of unknowns undermine the possibility to exactly reproduce the electrophysiological features of cat motoneuron sodium channels: (a) detailed voltage clamp studies of isolated Nav1.6 (and Nav1.2) channels heterologously expressed in (preferibly mammalian) cell systems exist for mouse or human sodium channels (which are in turn different), not for cats (Catterall et al., 2014); (b) it is neither exactly known the compartmental distribution in cat motoneurons of the different isoforms of sodium channels; (c) nor their fractional contribution to the global sodium currents is known. Given these limitations, we chose to implement in our models a previously described model of sodium channel (Powers et al., 2012), which proved useful to reproduce many of the experimental electrophysiological behavior of cat motoneurons.

However, due to the poor alignment of the fast and persistent sodium current as originally set, we modified the hemi-activation voltage of the gating variables of the two current in order to obtain a more plausible electrophysiological behavior of the current-voltage relationships. In particular, we shifted the hemi-activation voltage of the gating variables for the persistent current toward more depolarized values (+15 mV) and that for the fast current toward more hyperpolarized values (−5 mV) (see Supplemental Material).

Moreover, we set the persistent sodium conductance at a value able to produce a proportion of persistent current of about 20% compared to the peak of the transient one (Burbidge et al., 2002).

***Medium and distal dendrites***. The distal parts of the dendritic tree have been modeled with the lowest densities of the same conductances present at the soma, according to previous *in silico* studies (Powers et al., 2012). Moreover, in the dendritic branches far from the soma between 30 and 60% of the average dendritic length, a low-threshold Ca^2+^ conductance has been set to take into account the dendritic persistent inward current. Ca^2+^-activated K^+^ channels have also been uniformly distributed along these same regions of the dendrites.

***Axon hillock and AIS***. The axon hillock and the AIS include conductances modeling non-linear fast Na^+^, persistent Na^+^, and delayed rectifier K^+^ channel as well as a linear leakage conductance (Table 3). At the axon hillock, by varying the respective densities, the active channels of soma progressively replace those of the initial segment section, so that a gradual transition between active conductances has been set.

***Myelinated axon***. The nodes of Ranvier bring the parallel combination of non-linear fast Na^+^, persistent Na^+^, and slow K^+^ conductances, a linear leakage conductance, and the membrane capacitance (Table 4).

The paranodal and internodal compartments consisted of two layers, each including a linear conductance in parallel with the membrane capacitance, representing the myelin sheath and underlying axolemma.

### Simulation protocol

Due to the presence of active yet small conductances of the ionic channels at the depolarization starting value (−70 mV), few milliseconds were necessary to settle down a stable equilibrium potential, which slightly varied through the different sections. Thus, each simulation starts from a previously saved steady-state (300 ms without perturbations).

An integration time-step of 0.025 ms was adopted for all the simulations.

## Results

### Basal electrophysiological behavior of the models

**Figure 2:**
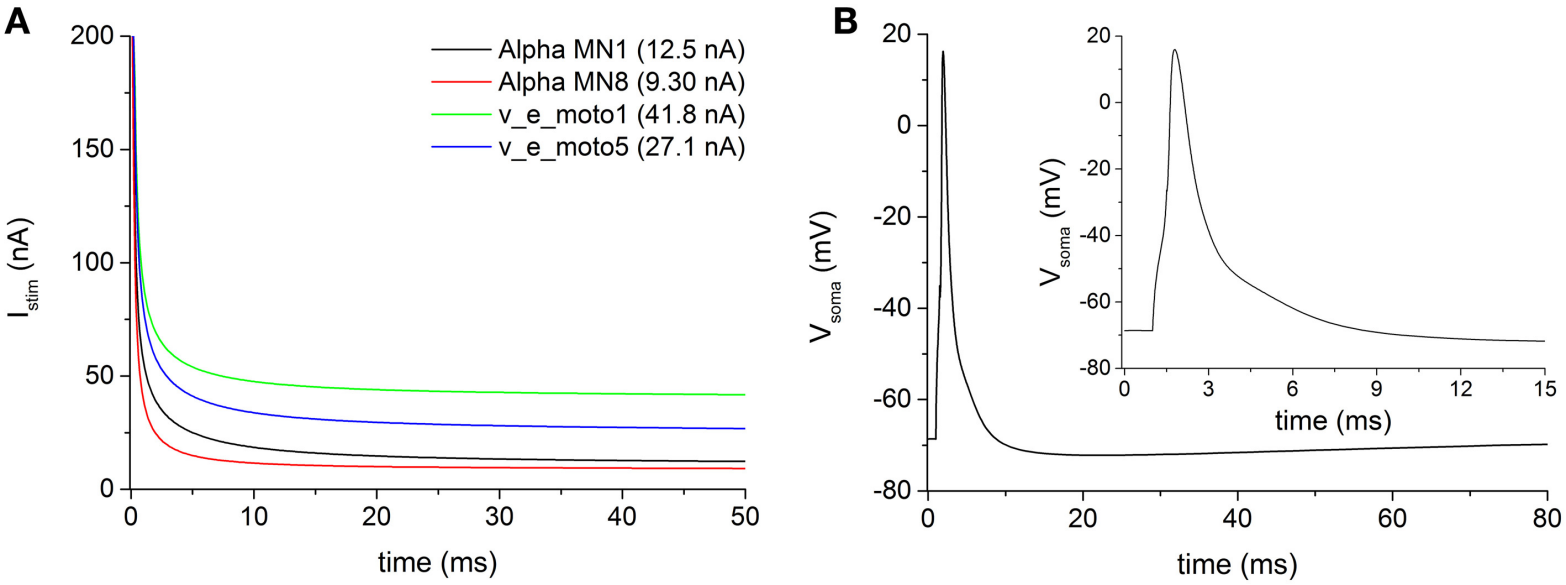
**(A)** Examples of stimulus intensity-duration curve, for measuring the rheobase, in four representative motoneurons of the pool. **(B)** Action potentials following a brief (0.5 ms) somatic depolarizing current recorded from soma of the motoneuron v_e_moto5. The inset shows the first 15 ms of the induced action potential.

The electrophysiological features of the simulated models are summarized in Table 5. The rheobase (minimal intensity of an infinitely long stimulus able to evoke a spike) was evaluated with a stimulus duration up to 1 s. Figure 2A shows four examples of stimulus intensity-duration curve relative to four alpha motoneuron models of different size.

**Table 5:** **Basal electrophysiological features of the neuron model pool**.

	**Rest potential (mV)**	**Rheobase (nA)**	**Suprathreshold stimulus (nA)**	**Peak latency (from stimulus, ms)**	**Peak amplitude (mV)**	**Maximal AHP amplitude (mV)**	**Maximal AHP latency (ms)**	**Z_IN_ at 0.1 Hz (MOhm)**	**Z_IN_ at 100 Hz (MOhm)**
AlphaMN1	−67.0	9.3	90	0.86	12.6	−4.8	22.6	0.47	0.33
AlphaMN2	−68.7	6.8	60	0.84	16.2	−4.3	19.3	0.89	0.66
AlphaMN3	−69.2	11.8	90	0.97	15.6	−4.1	17.5	0.60	0.47
AlphaMN4	−68.3	6.9	50	0.88	19.0	−4.3	20.5	0.81	0.60
AlphaMN5	−67.7	7.8	80	0.92	11.7	−4.4	21.3	0.58	0.41
AlphaMN6	−67.2	9.4	110	0.90	8.7	−5.0	21.3	0.47	0.32
AlphaMN8	−68.8	9.1	80	0.93	13.0	−4.2	18.2	0.74	0.56
AlphaMN9	−69.9	13.8	70	0.89	19.6	−3.2	18.6	0.63	0.53
v_e_moto1	−69.7	33.4	140	0.78	19.3	−2.6	20.4	0.26	0.21
v_e_moto2	−68.3	20.9	130	0.82	14.0	−3.5	22.1	0.32	0.24
v_e_moto3	−69.2	21.4	110	0.82	17.8	−3.0	21.3	0.40	0.31
v_e_moto4	−69.8	26.4	100	0.84	20.2	−2.5	20.8	0.31	0.27
v_e_moto5	−68.7	20.6	120	0.93	16.2	−3.5	21.0	0.31	0.24
v_e_moto6	−68.9	23.5	140	0.83	15.1	−3.3	21.1	0.29	0.23
Mean	−68.7	15.8	97.9	0.87	15.6	−3.8	20.4	0.51	0.38
SD	0.91	8.5	28.6	0.05	3.4	0.8	1.5	0.21	0.15

The modeled motoneurons predominantly belong to cells of big dimension (considering an average soma diameter of about 50 μm in alpha motoneurons). The increased motoneuronal size, especially in motoneurons from the second pool (i.e., v_e_moto1 to v_e_moto6, Table 1), results in relatively low values of input impedance (Z_IN_) and high values of rheobase (Figure 2A, green and blue traces).

The duration of the stimulus adopted to evoke an action potential at the soma was set to 0.5 ms. Figure 2B shows an example of somatic spike relative to the motoneuron v_e_moto5, elicited by a stimulus at the soma. It can be noticed the entire after-hyperpolarization that lasts about 80 ms.

### Voltage attenuation from first node to AIS

**Figure 3:**
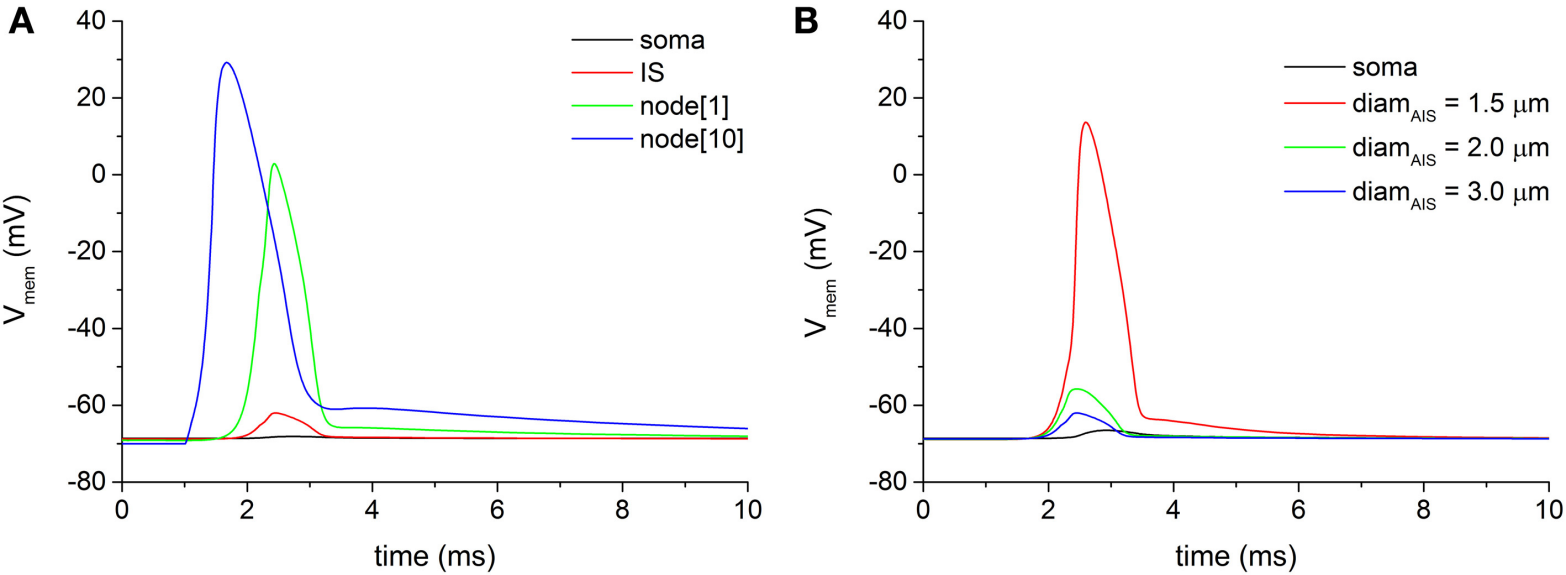
**(A)** Voltage attenuation encountered by the antidromically traveling wave at the transition regions between the first node of Ranvier (node[1], green trace) and the soma (black trace). Distal electric stimulation was applied at node[15]. **(B)** By decreasing the diameter of the axonal initial segment (IS), the voltage attenuation at the IS progressively lessens until the threshold for an action potential can be reached (red line).

We examined the antidromic propagation of the action potential from the myelinated axon through the AIS to the soma, following a distal stimulation of the axon. The distal node[15], located 17.25 mm far from the soma, was stimulated by means of a virtual electric pulse with a duration of 0.1 ms and an intensity of 5 nA. Given the extensive voltage attenuation that the antidromic traveling wave encounters along the axon-somatic junction, no antidromic spike is able to invade the soma in all neuron models bringing the basal biophysical and geometric parameters (Figure 3A). Figure 3A also shows a significant spike attenuation (mainly sustained by the dramatic geometric inhomogeneity of the axon-somatic junction), which already appears at the first node of Ranvier (green trace of Figure 3A), as well as the regularly conducted spike at the node[10] (blue trace of Figure 3A).

To overcome the wide voltage attenuation between the myelinated axon and the AIS, the geometric parameters of the AIS were swept. As Figure 3B shows, a reduction of the AIS diameter was able to decrease the attenuation at the point when the threshold for a spike is reached at the AIS. The reduced diameter, indeed, decreased the voltage attenuation for depolarization's approaching the AIS from the myelinated axon, due to a *sealed end* effect.

However, even with a fully delivered action potential at AIS, the voltage at the soma still remains well below the threshold (Figure 3B, black line), and, in each neuron model of the pool, no somatic antidromic propagation is possible. Therefore, it appears evident that two different sites of voltage attenuation are serially involved in preventing the antidromic somatic spike invasion: from myelinated axon to AIS and from AIS to soma.

### Voltage attenuation from AIS to soma

**Figure 4:**
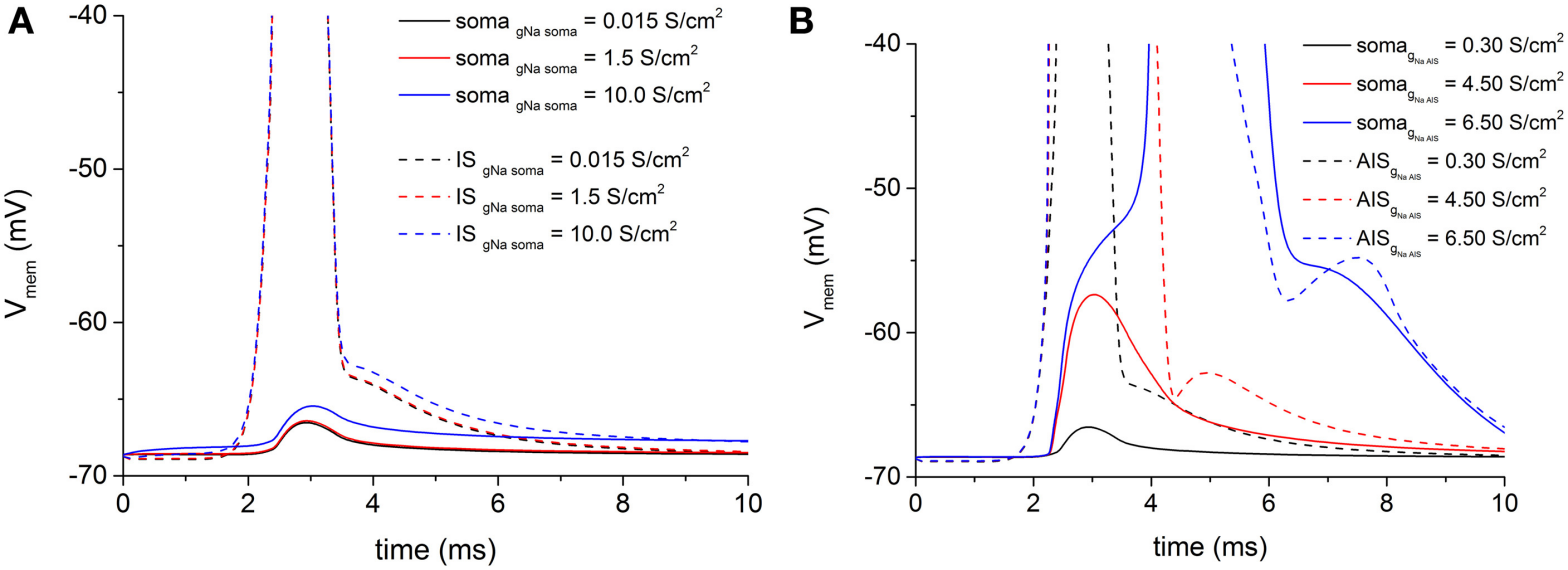
**(A)** Persistent voltage attenuation at soma (solid lines) blocks antidromic somatic spike propagation, notwithstanding increasing values of somatic fast Na^+^ conductance. As in Figure 3, an antidromic spike at IS (dashed lines) regularly develops for all Na^+^ conductance values. Basal Na^+^ maximal conductance at soma = 0.015 S/cm^2^. **(B)** The increase of fast Na^+^ channels conductance at the IS (dashed lines) is able to overcome the AIS-to-soma voltage attenuation and permits the antidromic spike propagation to the soma (solid lines). Basal Na^+^ maximal conductance at IS = 0.3 S/cm^2^.

As we arbitrarily chose the geometric AIS parameters, we have been able to sweep them in a realistic range to search for possible values which allow the spike antidromic propagation from myelinated axon to AIS. Regarding the voltage attenuation from AIS to soma, given the constraint of the imported and fixed morphological values of soma and dendrites, we needed a different way to facilitate the antidromic somatic spike invasion. Then, we increased the density of the somatic inactivating fast Na^+^ channels, because they are directly responsible for the spike initiation. However, even with extremely large increments of channels density at soma with respect to the initial conductance value, no soma invasion occurred (Figure 4A, red and blue solid lines). Values of Na^+^ channel density at soma higher than 10 S/cm^2^ induced a spontaneous and sustained depolarization of the motoneurons, not consistent with the experimental data.

On the contrary, when the density of the same channels at the AIS was increased to overcome the capacitive load of the soma and dendrites, it was sufficient to reach about 20-fold increments of the basal density to obtain a somatic spike (Figure 4B blue solid line).

Therefore, our simulations showed that changes in geometric and biophysical parameters of the AIS were necessary and sufficient to overcome the antidromic propagation failure both at the myelinated-unmyelinated axon and at AIS-soma transitions.

Table 6 shows a set of minimal values for AIS diameter and Na^+^ conductance in each neuron model able to permit the antidromic soma spike propagation.

**Table 6:** **Values of AIS parameters able to make the soma invasion of antidromic action potential possible**.

	**AIS diameter (μm)**	**Fast Na^+^ conductance at AIS (S/cm^2^)**
AlphaMN1	2.0	2.2
AlphaMN2	1.9	1.2
AlphaMN3	1.9	2.0
AlphaMN4	1.8	1.3
AlphaMN5	1.9	2.1
AlphaMN6	2.0	2.6
AlphaMN8	1.9	1.7
AlphaMN9	1.8	1.8
v_e_moto1	2.2	3.5
v_e_moto2	2.2	3.0
v_e_moto3	2.0	3.0
v_e_moto4	2.1	3.0
v_e_moto5	2.2	3.0
v_e_moto6	2.2	3.4
Mean	2.0	2.4
SD	0.15	0.76

### Voltage attenuations and the electrotonic distance

The myelinated axon-to-AIS and the AIS-to-soma voltage attenuations are principally sustained by the dramatically increased capacitive load downstream the antidromically traveling wave.

Based on the passive electrical properties of an ideal conductor, along infinite cables of uniform geometry the stationary voltage attenuation follows the well-known exponential cable equation:

(1) $${\text{V}}_{x}={\text{V}}_{0}e^{-\frac{x}{\lambda }}$$

with λ, the length constant, depending on the diameter of the cable conductor:

(2) $$\lambda =\sqrt{\left(\frac{R_{m}}{R_{i}}\right)\left(\frac{d}{4}\right)}$$

where *R*_*m*_ is the resistance across a unit area of passive membrane, *R*_*i*_ the volume resistivity of the intracellular medium, and *d* the diameter of the cable.

However, due to the wide difference between real neurites and an ideal cable conductor, and to the changing *R*_*m*_ in different regions of the neurons, according to previous studies on the measure of the electrotonic space (Carnevale et al., 1995) in real neurons, it can be useful to consider the electrotonic distance (*X* = x/λ) between two points in terms of voltage attenuation:

(3) $$\text{X}=ln\frac{V_{0}}{V_{x}}$$

By adopting the previous definition of electrotonic distance and by using the NEURON's tool for the study of electrotonus (Carnevale et al., 1995), it has been convenient to compute the different length constants in both the directions (ortho- and antidromic) of impulse propagation (Table 7).

**Table 7:** **Spatial constants and electrotonic distances in every neuron model, between AIS and soma and in both directions of impulse propagation**.

	**Soma > AIS λ (μm)**	**AIS > Soma λ (μm)**	**ln Attenuation Soma > AIS**	**ln Attenuation AIS > Soma**
AlphaMN1	581.2	45.0	0.114	1.471
AlphaMN2	538.8	64.2	0.116	0.973
AlphaMN3	582.5	53.0	0.114	1.253
AlphaMN4	549.7	59.2	0.115	1.067
AlphaMN5	571.9	50.5	0.114	1.290
AlphaMN6	578.6	46.6	0.114	1.413
AlphaMN8	541.4	58.0	0.115	1.107
AlphaMN9	591.4	52.3	0.114	1.287
v_e_moto1	714.3	37.3	0.112	2.144
v_e_moto2	653.5	38.8	0.113	1.903
v_e_moto3	660.2	39.6	0.113	1.884
v_e_moto4	690.2	38.9	0.112	1.985
v_e_moto5	658.4	39.2	0.113	1.899
v_e_moto6	671.2	38.6	0.113	1.962
Mean	613.09	47.23	0.114	1.55
SD	59.13	9.02	0.001	0.40

Table 7 shows how much the length constants between two points with high geometric non-uniformity do change depending on the direction of current flow, a longer length constant implying a more favorable progression of the spike.

As already described (Goldstein and Rall, 1974; Joyner et al., 1980; López-Aguado et al., 2002), indeed, at the site of an abrupt increase of diameter, significant changes happen both in voltage and ionic and axial currents. When the antidromic wave approaches the non-uniform region of the axon-soma junction, it must charge a larger capacity through a reduced axial resistance. As the action potential comes closer to the abrupt increase in diameter, its ability to provide the depolarizing current begins to be strained by the heavier load, resulting in a slower rise of the action potential and an attenuated amplitude. Even little changes of action potential amplitude cause an abnormally elevated sodium influx, because the driving force (*V* − E_Na_) remains large, which in turn can provide additional charge for depolarizing the adjacent region of increased diameter. However, as the axial current progressively increases, even the extra charge from the elevated inward sodium flux can become insufficient to depolarize the soma or the AIS membrane to threshold level.

## Discussion

In this study, we modeled the antidromic propagation of the action potential along a single motor fiber from the myelinated axon through the unmyelinated AIS to the soma of a spinal alpha motoneuron. To this purpose, we used a pool of morphologically detailed models of alpha motoneuron, exploiting the open-access web-based database of three-dimensional somato-dendritic reconstructions (www.neuromorpho.org) (Ascoli et al., 2007).

In addition, we adopted a highly detailed model of myelinated axon (McIntyre et al., 2002), which provided a realistic saltatory conduction in the myelinated part of the model. By using initial Na^+^ conductances similar to those adopted in previous *in silico* studies (Dodge and Cooley, 1973; Traub, 1977; Fleshman et al., 1988; Booth et al., 1997; Safronov et al., 2000; McIntyre et al., 2002; ElBasiouny et al., 2005; Shapiro and Lee, 2007; Cisi and Kohn, 2008; Powers et al., 2012) (Table 8), we unexpectedly found that in all neuron models no antidromic spike propagation occurred neither at the soma nor at the AIS.

**Table 8:** **Values of maximum conductance of fast inactivating Na^+^ channels in AIS and soma of previously developed models of alpha motoneurons in vertebrates**.

**References**	**AIS maximum fast Na^+^ conductance (S/cm^2^)**	**Soma maximum fast Na^+^ conductance (S/cm^2^)**
Dodge and Cooley, 1973	0.6	0.07
Traub, 1977	0.6	0.2
Fleshman et al., 1988	–	0.03
Booth et al., 1997	–	0.12
Safronov et al., 2000	1.32	0.002
McIntyre and Grill, 2002	0.5	0.05
ElBasiouny et al., 2005	1.34	0.06
Shapiro and Lee, 2007	0.005	0.006
Cisi and Kohn, 2008	–	0.03
Powers et al., 2012	0.55	0.044

### Does the somatic invasion of an antidromic traveling wave always occur in real neurons?

In real intracellular recordings, a failure of the antidromic somatic invasion represents an exception, and is often the result of experimental artifacts, like an induced somatic hyperpolarization or a repetitive antidromic stimulation (Eccles, 1955). In these cases, a subthreshold depolarization at soma is usually recorded and called NM (i.e., non-medullated) spike, according to the assumption that it is related to an action potential at the AIS. The antidromic spike propagation, in other words, reaches the AIS but fails to depolarize to the threshold the soma.

### Too big cells for somatic invasion to happen?

It can be hypothesized that spontaneous not induced blockages of antidromic spike mainly happen in motoneurons of larger size, due to the greater capacitive load provided by their soma and dendrites. Thus, the prevalence of large motoneurons in our pool of neuron models could bias our results toward a failure of the somatic invasion. As established in previous studies (Zengel et al., 1985), the relatively high values of rheobase of our pool, as well as the low ones of the input impedance, are in agreement with this dimensional bias. However, the failure of propagation was also detected in neuron models of the pool of smaller size. In addition, in every neuron model in basal configurations we were barely able to see even a slight depolarization at the soma following the antidromic spike propagation, which is in contrast with a main role of the excessively high neurons size in explaining our results.

### How many sites of dramatic voltage attenuation?

Our models provide for the first time a neurocomputational validation of a classical hypothesis carried out by the early electrophysiological studies, i.e., the probable failure of the antidromic propagation at the transition zone between the myelinated axon and the AIS (Eccles, 1955), in addition to the one occurring at the junction between AIS and soma. A substantial voltage attenuation, indeed, can be observed at these sites, mainly as a result of their marked geometric inhomogeneity.

### Biophysical significance of the study

Our study clearly shows that, when dealing with detailed morphological reconstructions of motoneurons, a much higher density of Na^+^ channels at the AIS has to be hypothesized to make the antidromic soma invasion to happen. Given that a precise and direct measure of Na^+^ channels density at the AIS of spinal motoneurons is still lacking, our hypothesis represents a neurocomputational prediction, which can be confirmed or contradicted by future experimental data.

Our findings show that the Na^+^ channels density at AIS should be set much higher than at the soma (Table 6). These data are in agreement with those of a previous study (Kole et al., 2008), which explored the densities of the Na^+^ channels at AIS and at the somato-dendritic compartment in cortical layer 5 pyramidal neurons by means of whole-cell patch clamping recordings, immunocytochemical imaging and neurocomputational models. That study found a value for Na^+^ channels density at AIS ~50 times higher than at the soma.

### The AIS

The AIS is a highly organized cellular domain specialized to function as the site of spike initiation in neurons (Clark et al., 2009). Its geometric and biophysical properties greatly influence the spiking features of the neurons. In addition, the segregation of ionic channels and their isoforms in different AIS subregions may explain a key role of the AIS in regulating the neuronal spike timing and frequency (Clark et al., 2009). In particular, a differential clustering of Na_v_1.2 and Na_v_1.6 sodium channel subtypes in the proximal or the distal region, respectively, of the AIS has been considered as the basis of two distinct tasks of the AIS: spike initiation and action potential back-propagation (Hu et al., 2009). The lowest threshold for spike initiation at the distal AIS should be determined by the high density of low-threshold Na_v_1.6 channels, while the high-threshold Na_v_1.2 channels control spike back-propagation because of their high density at the proximal AIS.

Although the present study did not adopt distinct subtypes of fast sodium channel, our findings are in agreement with this unique role of the AIS in spike back-propagation.

In our models, we directly attached the distal end of the AIS to the proximal edge of the first myelin attachment segment, so that the transition between these two segments was similar to that between a node and the myelin attachment segments. This agrees with the discovery that the axonal region where the myelin sheath begins seems organized with para-AIS and juxtapara-AIS, similar to the nodal regions (Duflocq et al., 2011).

### Detailed models

In this study, the development of detailed models of spinal motoneurons has provided novel insights about the voltage attenuation in proximal segments of the axon. Some experimentally testable predictions about the values of the densities of the Na^+^ channels at AIS and soma are also suggested.

Morphologically detailed models, indeed, have already demonstrated to provide more suitable results, compared to the reduced ones, especially when the examined events involve the integration of multiple synaptic inputs, or dendritic active conductances, or somato-dendritic back-propagation (Hendrickson et al., 2011). In particular, somato-dendritic back-propagation is considered a key mechanism for coincidence detection and synaptic long-term plasticity in neurons, and has been modeled in different types of neurons (Lüscher and Larkum, 1998; Diwakar et al., 2009; Hay et al., 2011). In these studies, morphologically detailed models have been successfully developed to simulate the spike back-propagation from soma to dendrites.

The implementation and the use of high-detailed neuron models to characterize the back-propagation from the myelinated axon through the initial segment to the soma, has been studied only in few cases. López-Aguado et al. (2002) modeled a single CA1 pyramidal cell obtained from detailed morphometric studies to describe the subcellular and macroscopic currents during axon-somatic or synaptic impulse propagation. This experimental and modeling work was able to show how the somatic transmembrane currents are entirely different during backward and forward spike propagation, and how the main cell axis behaves as a totally different cable when the action potential propagate in one direction or another. As above showed, a similar dichotomy is also clear from our results.

More recently, Kole et al. (2008) solved the discrepancies between cell-attached or outside-out patch-clamp studies, and whole-cell voltage-clamp data, by demonstrating a much higher density of Na^+^ channels at AIS than at the soma. Also in this work the computational part of the study was carried out by implementing highly detailed morphological reconstructions of the examined neuronal type (Kole et al., 2008).

Herein an axon-somatic back-propagation modeling study was performed for the first time in detailed models of spinal motoneurons, and further evidence was provided about the role of higher than usually admitted values of Na^+^ channel density at AIS.

Finally, our study also supports the view that the electrophysiological behavior of detailed models may help to guide the process of complexity reduction: the detailed model can prevent modelers from adopting minimizing strategies carrying to reduced models incompatible with the experimental neuronal features.

### Conflict of interest statement

The authors declare that the research was conducted in the absence of any commercial or financial relationships that could be construed as a potential conflict of interest.
