# Supplementary material for: Axon-somatic back-propagation in detailed models of spinal alpha motoneurons
Source: Front Comput Neurosci. 2015 Feb 12;9:15. doi: 10.3389/fncom.2015.00015 (PMC4325909; doi:10.3389/fncom.2015.00015)
Supplement: Supplementary file 1 [file DataSheet1.DOCX]

***Supplementary Material***

**Axon-somatic back-propagation in detailed models of spinal alpha motoneurons**

**Pietro Balbi ^1^*, Sergio Martinoia ^2^, Paolo Massobrio ^2^**

^1^ Department of Neurorehabilitation, ‘Salvatore Maugeri’ Foundation, IRCCS, Scientific Institute of Pavia via Boezio, Pavia, Italy

^2^ Department of Informatics, Bioengineering, Robotics, System Engineering (DIBRIS), University of Genova, Genova, Italy

*** Correspondence:** Pietro Balbi, Department of Neurorehabilitation, ‘Salvatore Maugeri’ Foundation, IRCCS, Scientific Institute of Pavia via Boezio n. 28, Pavia, 27100, Italy
email: pbalbi@fsm.it

1. **Supplementary Data**

In each compartment of the model the membrane potential is calculated using the differential equation (1) governing the balance of currents flowing across the membrane capacitance (*C_m_*), the leak conductance (*I_L_*), to adjacent compartments (*I_coupling_*) and other ionic conductances (*I_ionic_*), together with injected current (*I_inj_*):

*C_m_ (dv/dt) = – I_L_ – I_ionic_ – I_coupling_ + I_inj_* (1)

The ionic currents of the model follow the general form:

*I_ion_ = g_ion_ (V_m_ - E_ion_)* (2)

where *g_ion_* is the maximum conductance for the individual ion channel multiplied by gating variables that range from 0 to 1 (Hodgkin and Huxley, 1952). The Ca^2+^ and voltage dependence of each gating parameter is given by

*dω/dt = α_ω_(1 – ω) – β_ω_ω = (ω_∞_ - ω)/τ_ω_* (3)

where *ω* represents the fraction of open channels, *ω_∞_* is the equilibrium state, and *τ_ω_* is the time constant for approaching the equilibrium state.

The time course and magnitude of the activation and inactivation parameters are given below. The membrane dynamics were derived to be representative of neural excitation at 36°C.

*Fast sodium current*

*I_Naf_ = g_Naf_ · m^3^ · h · s · (V_m_ – E_Na_)* (4a)

where *g_Naf_* is the maximum conductance, *m*, *h*, and *s* are the gating variables for activation, fast inactivation and slow inactivation, respectively, and *E_Na_* is the equilibrium potential for Na^+^ (+50 mV). The equation describing activation (*α_m_*) and deactivation (*β_m_*) are:

*α_m_ = [0.4 · (V_m_ + 30)]/[1 – e^{-(Vm +30)/7.2}^]* (4b)

*β_m_ = [0.124 · (-V_m_ - 30))]/[1 – e^{-(-Vm - 30)/7.2}^]* (4c)

*τ_m_ = 1/(α_m_ + β_m_)* (4d)

where if *τ_m_* < 0.02 ms, then *τ_m_* = 0.02 ms

*m_∞_ = α_m_ /(α_m_ + β_m_)* (4e)

Fast inactivation is described by the following equations:

*α_h_ = [0.03 · (V_m_ + 40)]/[1 – e^{-(Vm + 40)/1.5}^]* (4f)

*β_h_ = [0.01 · (-V_m_ - 40)]/[1 – e^{-(-Vm - 40)/1.5}^]* (4g)

*τ_h_ = 1/(α_h_ + β_m_)* (4h)

where if *τ_m_* < 0.5 ms, then *τ_m_* = 0.5 ms

*h_∞_ = 1/[1 + e^{(Vm + 45)/4}^]* (4i)

Slow inactivation is described by the following equations:

*α_s_ = 0.001 · e^{-(Vm + 85)/30}^* (4j)

*β_s_ = 0.0034/[1 + e^{-(Vm + 17)/10}^]* (4k)

*τ_s_ = 1/(α_s_ + β_s_)* (4l)

*s_∞_ = 0.4 + 0.6 · α_s_ /(α_s_ + β_s_)* (4m)

*Persistent sodium current*

*I_Nap_ = g_Nap_ · m · s · (V_m_ – E_Na_)* (5a)

*τ_m_ = 1 ms* (5b)

*m_∞_ = 1/[1 + e^{-(Vm + 37.3)/6.8}^]* (5c)

*α_s_ = 0.001 · e^{-(Vm + 85)/30}^* (5d)

*β_s_ = 0.0034/[1 + e^{-(Vm + 17)/10}^]* (5e)

*τ_s_ = 1/(α_s_ + β_s_)* (5f)

*s_∞_ = 0.4 + 0.6 · α_s_ /(α_s_ + β_s_)* (5g)

*Delayed rectifier potassium current*

The delayed rectifier current is governed by the following equations:

*I_Kdr_ = g_Kdr_ · n^4^ · (V_m_ – E_K_)* (6a)

with E_K_ = -77 mV

*τ_n_ = 1.2 + 11.9 · [e^{(Vm + 39)/5.5}^]/[1 + e^{(Vm + 39)/5.5}^]^2^* (6b)

*n_∞_ = 1/[1+ e^{-(Vm + 25)/20}^]* (6c)

*Calcium dynamics and Ca-dependent K currents*

Ca^2+^ dynamics are represented by a simple, first-order process representing influx through Ca^2+^ channels and decay with a single time constant:

*dCa/dt = ([Ca]_∞_ - [Ca])/ τ_Ca_ - i_Ca_/(2 · F · d)* (7a)

where *τ_Ca_* is the time constant of decay, *i_Ca_* is Ca^2+^ current density, *F* is Faraday's constant, and *d* is the depth of the inner shell around the Ca^2+^-dependent K channels. This depth is given a small value (0.1 μm) for the Ca^2+^-dependent K channels governing the AHP, to allow for a relatively fast activation of these channels by Ca^2+^ influx during APs, whereas it is given a relatively large value (10 μm) for the Ca^2+^-dependent K channels colocalized with Cav1.3 channels to produce the relatively slow activation of this current. The time constant of decay for this slow activated Ca^2+^-dependent K channels is also given a relatively large value to produce a slow decay (200 ms), whereas the value of *τ_Ca_* for the Ca-dependent K channels mediating the AHP is set to 20 ms.

The relation between Ca^2+^ concentration and the activation of Ca-dependent K channels is described by the following equations:

*I_KCa_ = g_KCa_ · n · (V_m_ – E_K_)* (7b)

*α_n_ = 0.1 · ([Ca]-[Ca]_∞_)^2^* (7c)

*β_n_ = 0.1* (7d)

*τ_n_ = 1/(α_n_ + β_n_)* (7e)

*n_∞_ = α_n_ /(α_n_ + β_n_)* (7f)

*Ca*^2+^ *currents*

Both the high-threshold Ca^2+^ current providing the Ca^2+^ for Ca-dependent K channel mediating the AHP and the low-threshold Cav1.3 current are simulated using a sigmoidal steady-state activation curve and a voltage-independent time constant:

*I_Ca_ = g_Ca_ · m · (V_m_ – E_Ca_)* (8a)

*m_∞_ = 1/[1+ e^{-(Vm - Vhalf)/Vs}^]* (8b)

The *E_Ca_* is set to +80 mV. The half-activation voltage (V_half_) is -30 mV for the high-threshold Ca chanel and -43 mV for the low-threshold Cav1.3 channel. The activation slope (V_s_) is 4 mV for the high-threshold channel and 6 mV for the low-threshold channel, and the time constants are 1 and 60 ms, respectively.

*HCN current*

The hyperpolarization-activated mixed cation current is also simulated using a sigmoidal steady-state activation curve and a voltage-independent time constant of 50 ms:

*I_h_ = g_h_ · m · (V_m_ – E_h_)* (9a)

*m_∞_ = 1/[1+ e^{(Vm + 75)/8}^]* (9b)

The equilibrium potential for the mixed cation current (*E_h_*) is set to -38.9 mV.
